# Personalised Nutritional Plan and Resistance Exercise Program to Improve Health Parameters in Celiac Women

**DOI:** 10.3390/foods11203238

**Published:** 2022-10-17

**Authors:** Alejandro Martínez-Rodríguez, Daniela Alejandra Loaiza-Martínez, Javier Sánchez-Sánchez, Jacobo Á. Rubio-Arias, Fernando Alacid, Soledad Prats-Moya, María Martínez-Olcina, Rodrigo Yáñez-Sepúlveda, Pablo J. Marcos-Pardo

**Affiliations:** 1 Department of Analytical Chemistry, Nutrition and Food Science, University of Alicante, 03690 Alicante, Spain; 2 Alicante Institute for Health and Biomedical Research (ISABIAL), 03010 Alicante, Spain; 3 Carrera de Medicina, Facultad de Ciencias de la Salud, Universidad Indoamérica, Ambato 180151, Ecuador; 4 School of Sport and Science, European University of Madrid, 28670 Madrid, Spain; 5 Health Research Center (HUM628-Research Group), University of Almería, 04120 Almería, Spain; 6 Department of Education, Faculty of Educational Sciences, University of Almería, 04120 Almería, Spain; 7 Faculty of Education and Social Sciences, Universidad Andres Bello, Santiago 7550000, Chile; 8 SPORT Research Group (CTS-1024), CERNEP Research Center, University of Almería, 04120 Almería, Spain

**Keywords:** nutrition, gluten-free diet, physical activity, body composition, psychology

## Abstract

Celiac disease (CD) is a permanent immune reaction to gluten in those with a genetic predisposition. This study was designed to evaluate menopause-associated symptoms, mood, bone quality, and IgA antibody levels in women with CD, untreated and treated with a gluten-free diet (GFD), and with or without resistance exercise. The randomised controlled trial was conducted on 28 Spanish women (>40 years old). Participants were divided into the following intervention groups: personalised gluten-free nutrition plan + exercise (GFD + E); personalised gluten-free nutrition plan (GFD); celiac controls (NO-GFD); and non-celiac controls (CONTROL). The participants responded to the Menopause Rating Scale and the Profile of Mood States (POMS) questionnaires. Bone quality was measured with ultrasound and IgA with a blood test. After 12 weeks of intervention, the GFD + E group showed significant improvement in urogenital symptoms and scored higher on the ‘vigour’ subscale of the POMS. Negative associations were found between the total score on the Menopause Rating Scale and the ‘vigour’ subscale of the POMS questionnaire. Only those women who underwent a personalised GFD nutritional intervention combined with resistance exercise demonstrated significant changes after the intervention.

## 1. Introduction

Gluten intolerance is a systemic alteration in the immune system that can develop at various stages of the life cycle in response to gluten ingestion in genetically predisposed individuals [1,2]. Celiac disease (CD) is defined as a type of chronic, permanent intolerance to the gluten protein. In genetically predisposed individuals, this protein causes severe lesions in the mucosa of the small intestine, resulting in the atrophy of the intestinal villi, which determines the inadequate absorption of the nutrients in food, with consequent clinical and functional repercussions [3]. The food sources of gluten include foodstuffs of various types. Gluten is present in cereals such as wheat, barley, rye, triticale (a hybrid of wheat and rye), spelt (hexaploid wheat), Kamut (tetraploid wheat subspecies), and probably oats.

The diagnosis of CD is based on clinical history, serology, and endoscopy with duodenal biopsy, the latter of which is confirmatory for disease diagnosis. Several endoscopic features are described in CD, such as a loss of mucosal folds, a mosaic pattern, scalloped folds, nodularity, fissures, and the prominence of the submucosal vasculature. The sensitivity of upper endoscopy is close to 60% and the specificity is 95–100% [4,5]. It has been reported that the measurement of anti-tissue transglutaminase IgA and the total level of immunoglobulin A (IgA) in serum is the most cost-effective and accurate means of serological testing for CD [2]. This serological test is highly specific, sensitive, and less expensive than serum anti-endomysial antibody dosing. The IgA test measures the concentration of IgA, one of the body’s main antibodies, in blood.

The only treatment for CD is adherence to a strict gluten-free diet (GFD), which results in the disappearance of symptoms, the normalisation of serology, and the recovery of the intestinal villi. However, it should be kept in mind that many celiac patients following a GFD have nutritional deficiencies. In a review by Giorgia Vici et al. [6], it was observed that, in a general way, GFDs are poor in dietary fibre, in particular, due to the necessary avoidance of the several types of foods naturally rich in fibre (i.e., cereals) and the low fibre content of GF products that are generally made with starches and/or refined flours. They also found such diets to be poor in micronutrients, particularly vitamin D, vitamin B12, and folate, in addition to minerals such as iron, zinc, magnesium, and calcium. Inadequate macronutrient intakes were also reported, mostly related to a focus on gluten avoidance that often neglects the importance of nutritional quality. In fact, a higher content of saturated and hydrogenated fatty acids and an increase in the glycaemic index and glycaemic load of the meal were found. Therefore, an evaluation of the nutritional status of the celiac population and the nutritional quality of their diets is in order [7]. Nutritional imbalances among such patients should be detailed in depth, and adequate dietary guidelines should be offered for their correction, with the aim of improving the health and quality of life of this population.

Failure to follow a GFD diet can lead to major complications in celiac patients, which, especially in adulthood, can manifest themselves in the form of osteopenia, osteoporosis, and a high risk of neoplasms in the digestive tract, mainly in the gastrointestinal tract [1,8,9]. Although this treatment guarantees recovery from both the clinical symptoms and intestinal damage in almost all cases, it severely affects the patient’s quality of life [10]. In addition, in postmenopausal women, it is important to keep in mind that the long-term effects of oestrogen deficiency on the heart and bones lead to adverse cardiovascular changes and osteoporosis [11]. Low quality-of-life scores have been observed in women with menopausal symptoms [12].

Non-pharmacological interventions such as physical activity (PA) are among the effective methods of reducing menopausal symptoms, decreasing bone loss, and increasing muscle strength in menopausal women [13]. PA is defined as a behaviour that involves human movement, resulting in physiological attributes that include increased energy expenditure and improved physical fitness [14]. The benefits of PA are well-established; however, most middle-aged women are not physically active enough to meet physical activity guidelines, as women’s physical activity has been shown to decrease throughout their life cycle [15].

In this context, the aim of this investigation was to analyse the intensity of menopausal symptoms, mood, bone quality, and blood IgA antibody levels in adult women with CD undergoing different dietary and PA interventions. The initial hypothesis was that those celiac patients that did not follow a personalised GFD would have higher IgA antibody values, as well as poorer moods, because of the symptoms associated with untreated CD. It was also expected that the personalised diet intervention, together with a resistance exercise program, would improve the physical parameters and menopausal symptoms.

## 2. Materials and Methods

### 2.1. Subjects

In this investigation, 28 women (57.21 ± 11.41 years), perimenopausal and postmenopausal, 21 of whom were celiac, participated. All these participants were from Alicante, in the Valencian Community, Spain.

There were four intervention groups formed of seven women each. Perimenopausal (amenorrhea more or equal to 60 days but less than 1 year) and postmenopausal women (more than 1 year without menstruation) were eligible for inclusion in the study. The participants were asked questions about their menstrual cycle, regularity, and hot flashes. The Celiac Association of the Valencian Community (Celiac Association of the Valencian Community) was contacted for the dissemination of the research.

All those participants who suffered from any chronic disease related to the kidneys, thyroid, or heart; diabetes; or any psychological disorder were excluded from the research. Women were also excluded if they were taking oestrogens, had suffered a stressful situation, such as the death of a parent, during the last few weeks, were performing regular physical exercise, or were receiving treatment from a nutritionist at the time of participation. The participants were initially given an informative talk about the intentions, benefits, and commitment to the intervention.

### 2.2. Study Design

#### 2.2.1. Intervention

**Figure 1:**
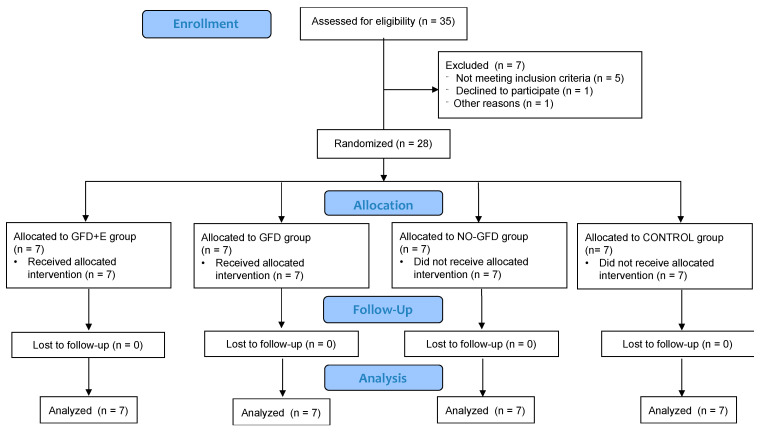
Consort 2021 flow diagram. Sample distribution.

A randomised clinical trial was conducted for 12 weeks. Four-block randomisation with a separate randomisation list of computer-generated random numbers was used to randomise the eligible participants. As shown in Figure 1, once enrolled, the subjects were submitted to the corresponding intervention: group 1, women with celiac disease following a personalised nutritional plan and resistance training (GFD + E); group 2, women with celiac disease following a personalised nutritional plan (GFD); group 3, women with celiac disease who were not receiving any type of intervention (NO-GFD); and group 4, healthy controls (CONTROL).

**Figure 2:**
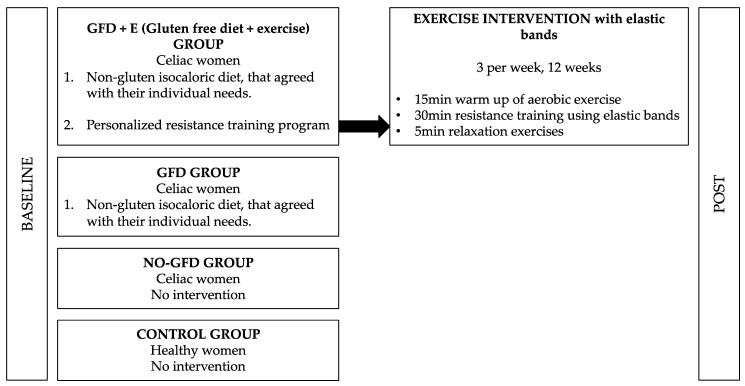
Different groups and interventions performed in the research.

Each of the groups received a different intervention, as shown in Figure 2. Group 1 (GFD + E) participants were instructed by a nutritionist to follow a personalised gluten-free isocaloric diet tailored to their individual needs. The macro- and micronutrient recommendations for the Spanish population were followed [16]. In this way, each participant had a plan adapted to her nutritional requirements and level of physical activity, paying special attention to gluten content. The Harris–Benedict equation was used, adjusting for the individual level of PA to calculate the resting specific energy expenditure (REE) [17]. All the patients were given a printed menu.

Following the American College of Sports Medicine’s recommendations, the participants underwent a customised resistance training program led by a graduate student in Physical Activity and Sports Sciences. All the patients in the training group attended all sessions. Resistance exercises for the major muscle groups were designed (Thera-Band^®^, The Hygenic Corporation, Akron, OH, USA). Both the intensity and the number of sets (from one to two) were progressively increased by changing the resistance of the bands (yellow–red–black). The Borg effort scale (from 1 to 10) was used to control the perception of effort after each training session.

Before the beginning of the investigation, the participants of group 2 with CD were independently following a GFD, not planned or directed by a nutrition specialist. The characteristics of groups 3 and 4 are shown in Figure 2. All the women completed the IPAQ questionnaire, with the aim of monitoring their daily physical activity. The differences between the groups were not significant.

This investigation was performed according to the standards of the Helsinki Declaration and received approval from the University Human Research Ethics Committee of Alicante University (Spain), code UA-2018-10-22. This trial was registered at clinicaltrials.gov (accessed on 27 October 2021) as NCT05052164.

#### 2.2.2. Measurement Tools

The data collection instruments included a demographic record sheet, the Menopause Rating Scale (MRS) to evaluate the presence of menopausal symptoms and their intensity, and the Profile of Mood States (POMS) self-report questionnaire for the measurement of mood, bone quality, and immunoglobulin IgA, at the time of the study and after 12 weeks of intervention.

##### The Menopause Rating Scale (MRS)

The MRS has three categories: physical, psychological, and urogenital. The subcategories include (1) physical (sweating/hot flashes, cardiac discomfort, sleep problems, and muscle and joint problems); (2) psychological (depressed mood, irritability, anxiety, and fatigue); (3) urogenital (sexual problems, bladder problems, and vaginal dryness). The internal consistency of the MRS questions was 0.83 according to Cronbach’s alpha, indicating the high reliability of the scale. The respondents were asked to choose from among five options: no symptoms, mild to moderate, marked, and severe. The total MRS score ranged from 0 (asymptomatic) to 44 (maximum degree of complaints). Based on our literature review, the total scores of ≤11, 12–35, and ≥36 are considered asymptomatic, mild to moderate, and severe to very severe, respectively. These 11 symptoms were then classified into 3 subgroups: somato-vegetative, psychological, and urogenital [12,18,19].

##### Profile of Mood States (POMS-29)

The abbreviated version of the Profile of Mood States (POMS-29) [20] in Spanish was used to assess mood and mood changes. This scale consists of 29 self-rated adjectives on a five-point scale ranging from 0 to 4 (not at all to extremely). The scale describes five mood states: tension, anger, vigour, fatigue, and depression. The questionnaire has been validated in a postmenopausal population [21], demonstrating the internal consistency and validity of the POMS for measuring the mood among postmenopausal women with moderate-to-severe hot flashes and the responsiveness of the POMS among those women with increased mood symptoms.

##### Bone Quality

An ultrasound heel densitometer (Achilles EXP II, GE Healthcare, Chicago, IL, USA) was used to measure each subject’s bilateral calcaneus. Quality control was performed by calibrating the device on a specific dummy provided by the manufacturer before the first measurement. An ultrasound gel medium was applied to ensure good contact. The speed of sound (SOS) and broadband ultrasound attenuation (BUA) were precisely measured during each ultrasonographic evaluation. The formula previously used in other studies [22], A.U. = (0.67 − BUA + 0.28 − SOS) − 420, was used to calculate the calcaneal stiffness (A.U.) index.

##### Blood Sample

Blood samples were collected to examine IgA levels. Reference values range from 43.63 mg/dL to 583.75 mg/dL, with a mean of 313.69 mg/dL [23]. IgA is one of the main antibodies in the body. In fact, the diagnosis of CD is based on the detection of highly specific serum IgA anti-transglutaminase, IgA autoantibodies, and the demonstration of duodenal villous atrophy [24]. This serological test is highly specific, sensitive, and less expensive than serum anti-endomysial antibody dosing [24].

### 2.3. Statistical Analyses

All statistical analyses were performed using Jamovi 1.1.3.0 software. Descriptive statistics were calculated (mean ± standard deviation). The Shapiro–Wilk test was used to test the normality of the distribution. Initial comparisons between the groups were performed using a one-way analysis of variance (ANOVA) followed by Tukey’s post hoc test, as appropriate. A group × time ANCOVA assay (using age as a covariate) was conducted, followed by a Bonferroni post hoc test, to assess the dissimilarities among the different evaluation times and treatments. Partial eta-squared (η2) effect sizes were calculated for time × group interaction effects. In addition, to establish the correlations between the variables of the study, Pearson’s correlation test was performed with 95% confidence intervals. The level of statistical significance was set at *p* ≤ 0.05.

## 3. Results

### 3.1. Baseline Characteristics

A total of 28 menopausal or postmenopausal women (57.21 ± 11.41; 41–74 years old; 161.6 ± 6.99; 148.7–171 cm height) took part in this study. There were significant differences between the different age groups (*p* < 0.001). Regarding BMI, the values were 26.2 ± 3.39 for group 1 (GFD + E), 27.9 ± 3.67 for group 2 (GFD), 24.6 ± 2.51 for group 3 (NO-GFD), and 29.3 ± 4.43 for group 4 (CONTROL).

### 3.2. Menopause Rating Scale (MRS)

**Figure 3:**
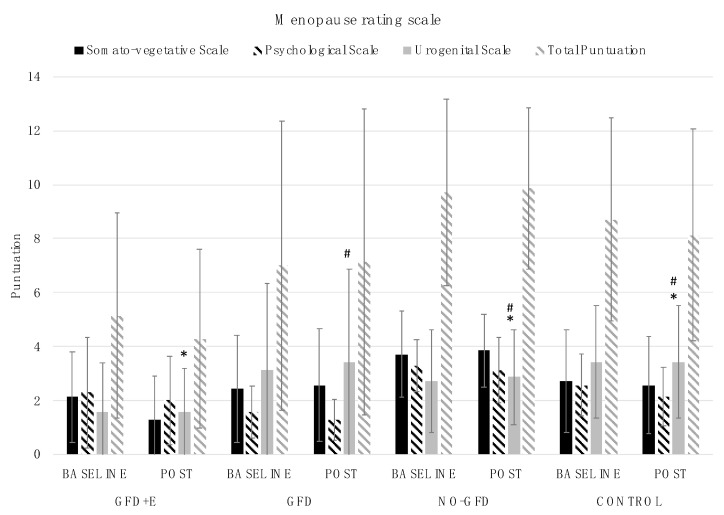
Descriptive statistics (mean ± standard deviation) of the Menopause Rating Scale: * differences between groups 1 (GFD+E), 3 (NO-GFD), and 4 (CONTROL); # differences between groups 2 (GFD), 3 (NO-GFD), and 4 (CONTROL); GFD + E, celiac women with a nutritional plan and physical exercise; GDF, celiac women with a nutritional plan; NO-GFD, celiac women with no nutritional plan or physical exercise; control, healthy women without diet or physical exercise intervention.

Figure 3 shows the total MRS results and the subscale data for each group. When age was used as a covariate, significant differences were observed in the urogenital scale scores (bladder problems, sexual problems, and vaginal dryness). Group 1 (GFD + E) presented significantly lower values (1.57 ± 1.6) than groups 3 (NO-GFD) (2.86 ± 1.77; *p* = 0.011) and 4 (3.43 ± 2.07; 0.013) at the postintervention time point. Significant differences were also found between group 2 (GFD) (3.43 ± 3.46) and group 3 (NO-GFD) (2.86 ± 1.77; *p* = 0.047) and between group 2 and group 4 (8.14 ± 3.93; *p* = 0.037) at the postintervention time point.

### 3.3. Profile of Mood States (POMS)

**Figure 4:**
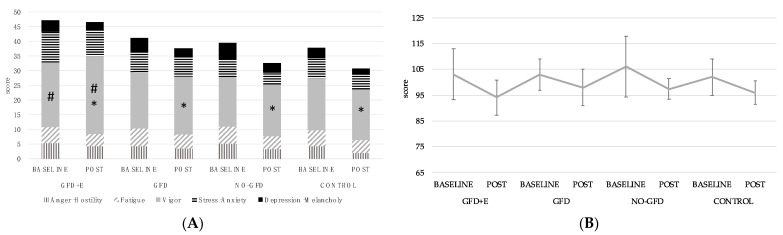
(**A**) Descriptive statistics of the different subscales of POMS questionnaire in menopausal and postmenopausal women participants; (**B**) descriptive statistics of the POMS total scores. POMS = Profile Of Mood States; * mean differences were significant at *p* < 0.005; # mean differences were significant at *p* < 0.001; GFD + E = celiac women with a nutritional plan and physical exercise; GDF, celiac women with a nutritional plan; NO-GFD, celiac women with no nutritional plan or physical exercise; control, healthy women without diet or physical exercise intervention.

The overall POMS score decreased significantly in all the groups (Figure 4B). The *p* value after performing a repeated-measure ANOVA was *p* < 0.001. However, following post hoc analysis, no remarkable differences were observed between the groups in the total score obtained. The same occurred in the rest of the subscales (Figure 4A); the *p* values after the effect–time analysis were *p* < 0.005.

Following the post hoc analysis, for the ‘vigour’ scale, significant differences in time were observed between the pre- and postintervention measurements of group 1 (*p* < 0.001). In addition, a significant increase was observed in the GFD + E group at the postintervention time point (26.3 ± 4.03) compared with the NO-GFD (17.7 ± 4.31; *p* = 0.004) and control (17.4 ± 2.76; *p* = 0.003) groups. Between the groups GFD + E and GFD (19.9 ± 3.34), there was a slight difference (*p* = 0.053). On the ‘tension’ scale, there was also a difference between the GFD + E (9 ± 3.21) and NO-GFD (4 ± 2.24) groups after the intervention (*p* = 0.056).

### 3.4. Bone Quality

Table 1 shows the SOS, BUA, and stiffness values measured with ultrasound of all the participants, separated by the intervention group. There were no significant differences observed in any of the groups.

### 3.5. Immunoglobulin A (IgA)

**Figure 5:**
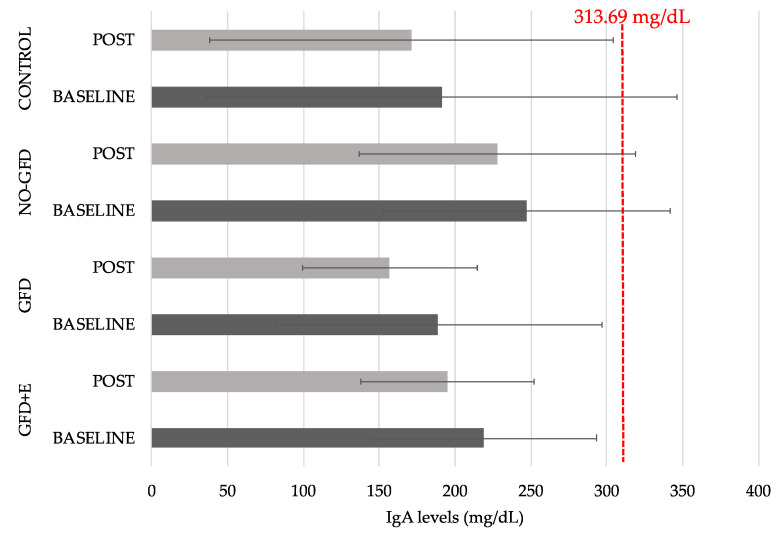
Descriptive statistics of blood IgA levels before and after intervention: GFD + E, celiac women with nutritional plan and physical exercise; GDF, celiac women with nutritional plan; NO-GFD, celiac women without nutritional plan or physical exercise; control, healthy women without dietary intervention or physical exercise. Reference values range from 43.63 mg/dL to 583.75 mg/dL, with a mean of 313.69 mg/dL [23].

Figure 5 shows the statistical summary of the blood IgA results. No significant differences were observed between the groups. It can be seen that the NO-GFD group had the highest values at the postintervention time point. The GFD group appeared to have the greatest difference between pre- and postintervention time points (189 ± 108 vs. 157 ± 57.4); however, these differences were not significant.

### 3.6. Correlations

Regarding the correlations (Table 2), a notable positive relationship was observed between age, the total score (*p* < 0.001), and the different subscales of the MRS; the higher the age, the higher the scores and, therefore, the greater the menopausal symptoms. Furthermore, age was also significantly and negatively related to the ‘vigour’ (*p* = 0.002) and ‘stress–anxiety’ (*p* = 0.032) subscales of the POMS questionnaire, as well as to the variable ‘stiffness index’ (*p* = 0.032). This suggests that the older the age, the lower the stress and bone stiffness and, therefore, the higher the risk of fracture. There was also a negative relationship between the ‘somato-vegetative scale’ (*p* = 0.001) and the ‘total’ score on the MRS questionnaire (*p* = 0.017) with the ‘vigour’ scale of the POMS.

## 4. Discussion

This study evaluated the impact of different GFD and PA interventions on menopausal symptoms, mood, bone quality, and blood IgA antibody levels in post- and perimenopausal adult women in a 12-week randomised controlled trial. Overall, the only group that showed significant differences in mood variables after the intervention was the group that followed a personalised GFD diet + resistance exercise program throughout the 12 weeks. Moreover, after the intervention, there were significant differences between the groups in terms of menopausal symptoms referring to the urogenital subscale, including differences between GFD + E and NO-GFD, GFD and NO-GFD, and GFD and control.

In addition to the classic gastrointestinal (GI) symptoms, extraintestinal symptoms, including neurological, psychiatric, and skin-related symptoms of CD, are increasingly recognised. A complex interaction between CD and these psychiatric disorders is proposed in the literature [25]. Theories are often divided into specific and non-specific mechanisms [26]. Specific mechanisms refer to those biological processes that may be producing overlapping pathologies, such as speculation about a direct ‘intestinal–non-brain’ relationship [27,28]. Non-specific mechanisms include the social and emotional consequences of CD diagnosis [29].

Research in post- and perimenopausal celiac women is quite limited, making it difficult to compare the results from different studies on the same population. In a study involving 114 patients with CD, symptomatic patients reported a relative improvement in their quality of life following a GFD, compared with that at diagnosis [30]. In this sense, it seems that following a GFD improves well-being.

However, in any studied population, it should be noted that in addition to all the symptoms and disorders typical of CD, the symptoms typical of menopause and perimenopause should also be considered, including hot flashes and night sweats, insomnia, vaginal dryness, and mood disorders [31], as well as the risk of developing depression or anxiety disorders [32]. Although these symptoms are not life-threatening, they can actually have an adverse effect on women’s quality of life and physical and mental health [33].

There is no research on celiac women analysing these symptoms and disorders. However, similar to the findings obtained in the present investigation, a cross-sectional study in women aged 40–65 years observed differences depending on the PA level: Moderately active women had less severe and moderate symptoms than inactive women [13], both in urogenital symptoms (*p* = 0.046) and in total scores (*p* = 0.019). In a cross-sectional, analytical study involving 190 postmenopausal women aged 40–64 years [34], as the participants increased their level of PA, their total scores on the MRS and the psychological and urogenital subdimensions decreased.

In another 2-month investigation involving a PA intervention, a significant reduction in the frequency and severity of all menopausal symptoms, including hot flashes, sleep problems, and joint discomfort, was observed [35]. The results obtained in these two studies are in partial agreement: Although there were differences between the groups, there were no differences in any group before and after the intervention with GFD and resistance exercise. Our results show that the NO-GFD group obtained the highest score on the MRS, followed by the control, GFD, and GFD + E groups. It should be noted that, although the differences were not significant, in celiac women, the total score on the scale only decreased in the group that performed resistance training in addition to following a GFD.

There is strong evidence that physical inactivity increases the average levels of anxiety, stress, depression, and menopausal symptoms [36,37,38,39]. Since the same moderate level of physical activity could affect the overall health, it seems that the government and health centres could encourage women to engage in regular and planned physical activities by conducting training classes and appropriate programs, increasing women’s awareness, and creating a positive attitude towards this period [40].

It should be added that somatic and psychological symptoms were higher in the NO-GFD group; therefore, it seems that the group of celiac patients that did not follow a personalised GFD was the one with the most severe symptoms. For urogenital symptoms, the GFD and control groups scored the same; however, within the group of celiac women, the highest score was found in the GFD group (following a GFD but no physical exercise). In view of these results, it seems that, in celiac women, the fact of following a GFD does improve somatic and psychological symptoms; however, it is necessary to add PA so that urogenital symptoms are also improved.

Our observations agree with those of previous authors [13,41], who specified that PA is one of the independent components related to the severity of menopausal symptoms; they observed that moderately active women had reduced severe and moderate symptoms, compared with inactive women, in addition to better general well-being and health status [37,42]. Compared with other investigations [18], the women in the present investigation had lower total and subscale scores.

Regarding the possible association between mood disorders and gluten sensitivity, no systematic studies have been conducted. A higher rate of autoimmune diseases (such as CD) has been demonstrated in people with mood disorders; however, since they have not been systematically studied as disorders, their association with CD is neither conclusive nor systematic and is limited to depression, often accompanied by anxiety [43]. Our findings show that the GFD + E group was the only group that presented significant improvements in the ‘vigour’ dimension of the POMS; however, no significant changes were observed on the rest of the subscales of the questionnaire. The ‘cholera’, ‘fatigue’, and ‘depression’ scores at the baseline might have been too low to detect a significant reduction after 12 weeks. Therefore, among celiac women, there was no difference between those who followed the diet and those who did not.

Regarding bone quality in celiac women, studies have reported that dietary compliance with DLG adherence has a positive effect on bone mineral density [44]; however, these improvements in BMD may take 2–5 years [44]. Sustained PA has beneficial effects on the bone and works to attenuate bone loss [45]. Studies [46] conducted with postmenopausal women who exercised for 12 months or more have shown small increases in BMD. These reasons explain why none of the women in the present investigation significantly improved.

As for the IgA levels, none of the groups had elevated values. For this reason, no significant changes were observed after a GFD prescribed by a specialist was followed since prior to the study, they had all attempted to avoid gluten on their own.

In terms of correlations, a negative association was found between the ‘somato-vegetative’ subscale and the MRS total score on the ‘vigour’ subscale. The somato-vegetative domain includes hot flashes, cardiac discomfort, sleep difficulties, and muscular and joint discomfort; therefore, the higher the prevalence of these symptoms, the lower the scores on the ‘vigour’ scale, i.e., the lower the persistent mental and physical activation [36]. In addition, it was also observed that the older the age, the greater the menopausal symptoms and the lower the bone stiffness. These findings are not surprising, since it has been shown that with respect to BMD, approximately 10% of women aged 60 years, 20% of those aged 70 years, 40% of women aged 80 years, and 67% of those aged 90 years suffer from osteoporosis [47].

It should be noted that the adoption of a GFD has become increasingly popular in both North America and Europe, eclipsing fat-free and low-carbohydrate diets. These supposed ‘free-form diets’ are bombarding the media and social networks, and the market for GFDs is incredibly profitable [48]. Adopting a GFD without having gluten sensitivity can be harmful to the body if the correct balance of carbohydrates, proteins, and lipids is not applied. It has been established which pathologies can improve or worsen in symptomatic non-celiac subjects when adopting a GFD [48]. In those patients with endometriosis, fibromyalgia, irritable bowel syndrome symptoms, psychosis, and schizophrenia, it may have beneficial effects; however, for other pathologies such as fibre deficiency, hyperlipidaemia, hyperglycaemia, and micronutrient deficiency, among others, it may worsen the patient’s symptoms [48].

The strengths of this study include its RCT design, where four different groups (GFD + E, GFD, NO-GFD, and control) could be compared. Nevertheless, the study also had several limitations. They include the small sample size (although there are investigations that work with similar sizes [49]) since it is limited to a specific patient population (women with CD) and the cost of the tests. To measure bone mineral density, we used a calcaneal densitometer. The ideal option would have been to perform bone densitometry (dual-energy X-ray absorptiometry), but this method, considered the ‘gold standard’, was not practical due to its low accessibility and high economic cost. In future lines of research, the detection of serum anti-transglutaminase-2 (TG2) IgA should be performed, since it is a highly specific, sensitive, and less expensive serological test than serum anti-endomysial antibody dosing. In Spain, the prevalence of CD is higher in children (1:71) than in adults (1:357) [50], which makes it difficult to achieve larger study sample sizes, and there is a lack of diagnosis in many populations. It would be ideal to confirm these results with a larger group and over a longer period.

## 5. Conclusions

Following a GFD together with resistance training using elastic bands in post- and perimenopausal celiac women improves menopausal symptoms, quality of life, and mood compared with women who do not undergo training. Personalised gluten-free planning is not sufficient to observe changes in the studied variables. It needs to be complemented with physical activity. Adequate training and effective regular physical activity interventions may be important steps to promote the overall health of menopausal women. A longer intervention of both GFD and physical exercise is necessary to see changes in BMD.

## Figures and Tables

**Table 1:** Descriptive data on bone quality.

	GFD+E						GFD						NO-GFD						CONTROL					
	BASELINE			POST			BASELINE			POST			BASELINE			POST			BASELINE			POST		
Stiffness Index (A.U)	106	±	17.6	106	±	17.4	94.3	±	15.3	95.9	±	14.7	88.3	±	12.6	89.1	±	12.4	87.4	±	6.58	89.0	±	4.51
BUA (dB/MHz)	123	±	12.9	124	±	13.1	118	±	14.2	119	±	14.3	115	±	17.2	116	±	15.2	103	±	30.6	105	±	30.1
SOS (m/s)	1588	±	50.0	1588	±	49.9	1557	±	28.8	1562	±	29.4	1541	±	18.9	1543	±	22.6	1331	±	570	1334	±	571

Data in the table are shown as mean ± SD. SD: standard deviation; BUA: broadband ultrasound attenuation; SOS: speed of sound; dB = decibel-milliwatt; MHz = megahertz; m = meters; s = seconds.

**Table 2:** Correlations between the different variables analysed in the research.

	Age (years)	Height (cm)	BMI (kg/m^2^)	MRS S-V	MRS PSCHY	MRS UG	MRS TOTAL	POMS TOTAL	A-H	Fatigue	Vigour	S-A	D-M	SI (A.U)	BUA (dB/MHz)	SOS (m/s)	IgA
Age (years)	—																
Height (cm)	−0.647 **	—															
BMI (kg/m^2^)	0.145	−0.118	—														
MRS S-V	0.681 **	−0.323	0.057	—													
MRS PSCHY	0.186	−0.066	−0.113	0.383 *	—												
MRS UG	0.787 **	−0.391 *	0.235	0.674 **	0.152	—											
MRS TOTAL	0.782 **	−0.393	0.149	0.899 **	0.539 *	0.851 **	—										
POMS TOTAL	0.185	−0.240	0.260	0.264	0.053	0.216	0.277	—									
A-H	−0.339	0.192	−0.060	−0.283	−0.310	−0.274	−0.359	0.051	—								
Fatigue	0.028	−0.022	0.328	0.104	0.197	0.081	0.174	0.614 **	0.086	—							
Vigour	−0.550 *	0.508 *	−0.162	−0.470 *	−0.147	−0.285	−0.447 *	−0.377	0.586 *	−0.034	—						
S-A	−0.406 *	0.300	0.044	−0.162	−0.022	−0.039	−0.136	0.371	0.105	0.151	0.458 *	—					
D-M	0.132	−0.073	−0.021	0.011	−0.075	0.160	0.070	0.622 **	0.379 *	0.320	0.148	0.294	—				
SI (A.U)	−0.410 *	0.497 *	0.059	−0.069	−0.035	−0.187	−0.132	0.114	0.306	0.249	0.335	0.328	0.064	—			
BUA (dB/MHz)	−0.192	0.309	−0.029	0.013	−0.319	−0.176	−0.177	0.119	0.276	0.035	0.114	0.023	0.289	0.561 *	—		
SOS (m/s)	−0.150	0.018	−0.363	0.029	−0.274	−0.203	−0.187	0.009	0.221	−0.136	0.044	−0.129	0.287	0.173	0.739 **	—	
IgA	0.115	0.245	0.088	0.173	0.161	0.216	0.252	0.186	0.110	0.196	0.064	0.175	0.092	0.205	−0.276	−0.574 **	—

* *p* < 0.05, ** *p* < 0.001; cm = centimetres; kg = kilograms; m = meters; MRS = Menopause Rating Scale; S-V = somato-vegetative; A-H = anger–hostility; S-A = stress–anxiety; D-M = depression–melancholy; PSCHY = psychological; UG = urogenital; SI = Stiffness Index; BUA: broadband ultrasound attenuation; SOS: speed of sound; dB = decibel-milliwatt; MHz = megahertz; m = meters; s = seconds.

## Data Availability

The data presented in this study are available on request from the corresponding author. The data are not publicly available as they concerned personal health information.
